# Sport and non-specific low back pain in athletes: a scoping review

**DOI:** 10.1186/s13102-022-00609-9

**Published:** 2022-12-23

**Authors:** Fulvio Dal Farra, Federico Arippa, Giuseppe Carta, Marco Segreto, Elisa Porcu, Marco Monticone

**Affiliations:** 1 Department of Medical Sciences and Public Health, University of Cagliari, Cagliari, Italy; 2 Neurorehabilitation Unit, Department of Neuroscience and Rehabilitation, ARNAS G. Brotzu, Cagliari, Italy; 3 Physical and Rehabilitation Medicine, Clinical Sciences and Translational Medicine Department, University of Rome Tor Vergata, Rome, Italy; 4 Nuova Sair, Rome, Italy; 5 Gabinetto di Fisiokinesiterapia, Villasor, Italy

**Keywords:** Sport, Low back pain, Epidemiology, Scoping review, Athlete, Risk factor

## Abstract

**Background:**

The contribution of sport in non-specific low back pain (NS-LBP) remains unknown, due to a large heterogeneity in the methods applied in research. The aims of this scoping review (ScR) were to systematically map and summarize findings concerning studies reporting data on NS-LBP among athletes.

**Methods:**

This ScR was developed referring to the 2020 version of the “Joanna Briggs Institute Methodological Guidance” and the Preferred Reporting Items for Systematic Reviews and Meta-Analyses for Scoping Reviews. Five medical databases (Pubmed, Cochrane, Central, Embase, Pedro and Scopus) were searched up to November 2021. No limitations in terms of study design and language were applied. Results were presented numerically and thematically.

**Results:**

A total of 4061 records were identified through the initial search; 114 articles met the inclusion criteria. Publications have increased over the years, since 1990. Most of the studies were conducted in the USA (17.5%), even if most research was conducted in Europe (53.5%). Analytic observational (42%) and cross-sectional studies (37%) were the most used designs, followed by case reports (12%) and systematic reviews (9%). Boating (7%), football, soccer, volleyball, running and gymnastics (4.4% each) were the most investigated, although the majority of the studies considered sports in general (36.8%). The overall sample size median was 181, mean age 22 ± 10.2; 68% of athletes were professional and 32% amateur. Most of the studies (38%) did not detail the frequency of training. Sport was reported as a risk factor for developing NS-LBP in 67.5% of cases, especially in those studies which assessed activities implying high or repeated loading on the spine.

**Conclusions:**

This is the first ScR to provide a comprehensive overview on this topic. The increased number of publications on the association between sport practice and NS-LBP demonstrates a growing interest over the years on this topic. Some sport activities seem to be more involved than others in LBP development; however, research methods are extremely varied, thus more standardized observational research may focus on specific disciplines to properly contribute to research and clinical practice.

**Supplementary Information:**

The online version contains supplementary material available at 10.1186/s13102-022-00609-9.

## Background

Low back pain (LBP) is considered as one of the most significant health problems worldwide, with a global prevalence of 38.9% [1]. LBP represents a multifactorial disease with several underlying causes such as occupational and psychological factors, age, gender and other social-demographic features, but also lifestyle and mechanical issues [2]. These aspects inevitably create an important burden to different degrees, including individual-, community- and economic-based difficulties [3]. Furthermore, a large amount of LBP can be defined as non-specific (NS-LBP), referring to a condition where an identifiable source of pain is not recognizable [4].

While most of the above-mentioned factors are generally acknowledged as risk conditions for NS-LBP, the impact of physical stress on the lumbar spine due to sports seems conflicting [5–8]. A recent meta-analysis suggests how leisure time physical activities moderately protect from the risk of developing NS-LBP [9], while there is inconsistent evidence in favor or against more intensive physical training [10, 11].

A previous literature search showed how several studies investigated a possible association between sport activities and NS-LBP, despite a wide variety of methods and results [6, 12]. Several differences were found in the retrieved studies, including population types, sport activities, characteristics of training, modalities of LBP assessments and risk analyses [6, 12]. In particular, studies differed for age range of the selected population, included disciplines (i.e., soccer, skiing, rowing, handball, volleyball, basketball, skating, hockey, tennis, golf, ballet, track, swimming, softball, orienteering) as well as for training frequency, pain definition, localization, intensity and duration; moreover, a number of methods was used for LBP identification. For such reasons, currently, it appears difficult that a systematic review investigating the impact of different sports on NS-LBP could lead to firm recommendations.

However, there is a need to highlight some general aspects on this topic, such as the main sport activities which the literature focuses on, the settings where they are performed, the sample characteristics or the training modalities (e.g., frequency, intensity, duration), as well as the study designs actually adopted. According to the Joanna Briggs Institute (JBI), a scoping review (ScR) represents the most useful approach to map literature and to clarify key concepts and possible shortcomings a specific research area may have [13]. Hence, such a comprehensive report may be interesting, in order to inform and provide more indications in sports’ practice for subjects with NS-LBP [13, 14].

As to our knowledge, a complete overview of NS-LBP among athletes is missing, the aim of this paper was to map the existing literature concerning the possible association between the practice of the main sports activities and LBP occurrence. Hence, the following research question was posed: “what is known from the current literature about the association between sports and LBP in athletes?”. In particular, the aims were as follows: (1) to undertake a ScR to systematically map and summarize the literature reporting epidemiological data on sport activities and NS-LBP; (2) to identify any possible shortcomings in knowledge concerning this topic; (3) to provide cues and suggestions for clinicians, researchers and stakeholders.

## Methods

### Registration and reporting

This scoping review was developed referring to the “2020 version of the Joanna Briggs Institute Reviewers’ Manual” [13] and the “Preferred reporting items for systematic reviews and meta-analysis extension for scoping reviews” (PRISMA-ScR) checklist [15]. The protocol was stored on the OSF with the following registration number: https://doi.org/10.17605/OSF.IO/9BEX8 [16].

### Inclusion and exclusion criteria

As recommended, studies were included when they met specific criteria in terms of population, concept and context [17]. Specifically, we considered original research dealing with athletes of any country (elite or amateur, male or female, adolescent or adult), who practice different types of sport. Studies focused on leisure-time physical activity were excluded. The concept was focused on the association between the sport activity and NS-LBP onset, in each possible context, such as professional teams, amateur clubs or colleges. As a consequence, we included only articles in which epidemiological indicators (e.g. relative risk, odds ratio, frequency distributions, incidence and prevalence) concerning the association “sport activity and NS-LBP” were present. Conversely, studies investigating only peculiar aspects, such as physical characteristics (e.g. flexibility, muscle size, physical performances, etc.) in relation to NS-LBP were excluded.

Examples of the sports considered are football, soccer, volleyball, basketball, tennis, running, golf and cycling, even if other types of activities were included if retrieved in the search results. Only observational studies (both analytical and descriptive) and systematic reviews were accepted, without any restrictions in terms of time, setting and country; narrative reviews, commentary and letters to editors were excluded. Only articles in English were accepted. We excluded studies considering some particular activities (such as car racing and fishing), where movement does not represent the central element in the potential association with NS-LBP.

### Search strategy

The literature search was carried out by consulting the main biomedical databases such as Pubmed (Medline), Central (Cochrane), Embase, Pedro and Scopus. We composed different query strings depending on the variability of the databases functioning; however, we always considered the following Mesh- or free-terms: sport*, athlet*, football, volleyball, tennis, basket*, running*, soccer, cycling, gymnastic*, low* back pain, spinal pain, backache, lumbago, non-specific low back pain, low back ache. Pubmed search query is reported in the Additional file 1: Appendix A.

Gray literature was considered via Google Scholar. In order to avoid missing any possible relevant source, additional records were periodically searched through cross-referencing. Literature was searched up from January 1990 to November 2021.

### Study selection and data extraction

The search strategy results were managed through “Rayyan – the intelligent systematic review” web app (www.rayyan.ai) [18].

**Fig. 1:**
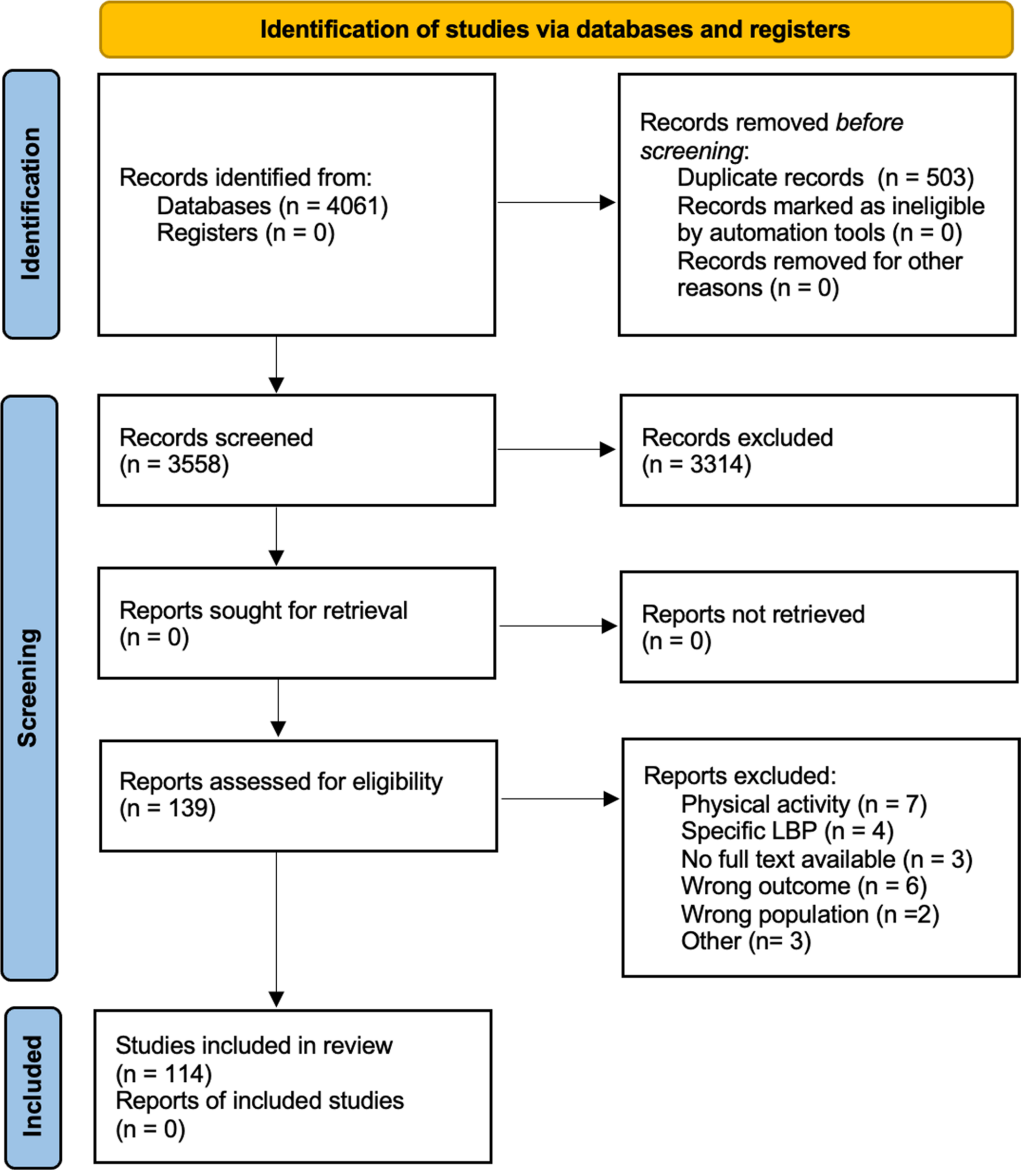
Flow diagram based on PRISMA statement (www.prisma-statement.org)

Duplicates were automatically deleted, and records were screened firstly by title and/or abstract and secondly by full-text reading. Two blinded authors (GC, MS) independently screened the articles, and any possible conflicts were resolved through a discussion with three expert authors (FDF, FA, and MM). Specific details of the selection process are better illustrated in the PRISMA flow diagram (Fig. 1). The main features of the included studies in relation to the aim of the review were reported in a data extraction form. This form was previously developed, discussed, implemented and accepted by all the authors of the study. The major characteristics extracted from the included works were: year of publication and country, type of journal (sport medicine journal or not), study aim, study design and duration, sample size, description of the sport activity (typology, rate of attendance, etc.). Finally, a summary of the main results was reported.

### Synthesis of results

Data were reported numerically and thematically. Descriptive statistics was used reporting means, standard deviation (SD), median, mode, interquartile ranges (IQR) and percentages for all the considered outcomes, such as period of publication, country, study design, characteristics of participants, sports activity and results. Results have been examined by the two blinded reviewers (GC, MS) and subsequently, specific thematic areas were detected through a discussion with the other expert authors (FDF, FA, MM).

Considering the large number of variables, we opted to report our results using several graphs in order to facilitate the reader with the interpretation of the results.

## Results

A total of 4061 articles were identified through the initial database search; 503 records were detected as duplicated and consequently removed. Overall, 3558 articles were screened for title and abstract and 3314 of them did not meet the inclusion criteria and were therefore rejected. Following the full-text reading, 24 records were excluded with reasons. As a result, 114 articles were definitively included in the qualitative synthesis. Further details concerning the study selection process are reported in the PRISMA flow diagram (Fig. 1).

### Study characteristics

Among the 114 included works, 104 (91%) belong to primary research (observational studies, both descriptive and analytical). Conversely, 10 (9%) are systematic reviews and/or meta-analyses. In detail, 48 articles (42%) are analytical studies (case–control or cohort), 42 studies (37%) consist of cross-sectional investigations, 13 (11.5%) are case reports and only 1 (< 1%) is a case-series. The distribution of study designs among the included works is graphically reported in Additional file 2: Fig. B1 (Appendix B).

**Fig. 2:**
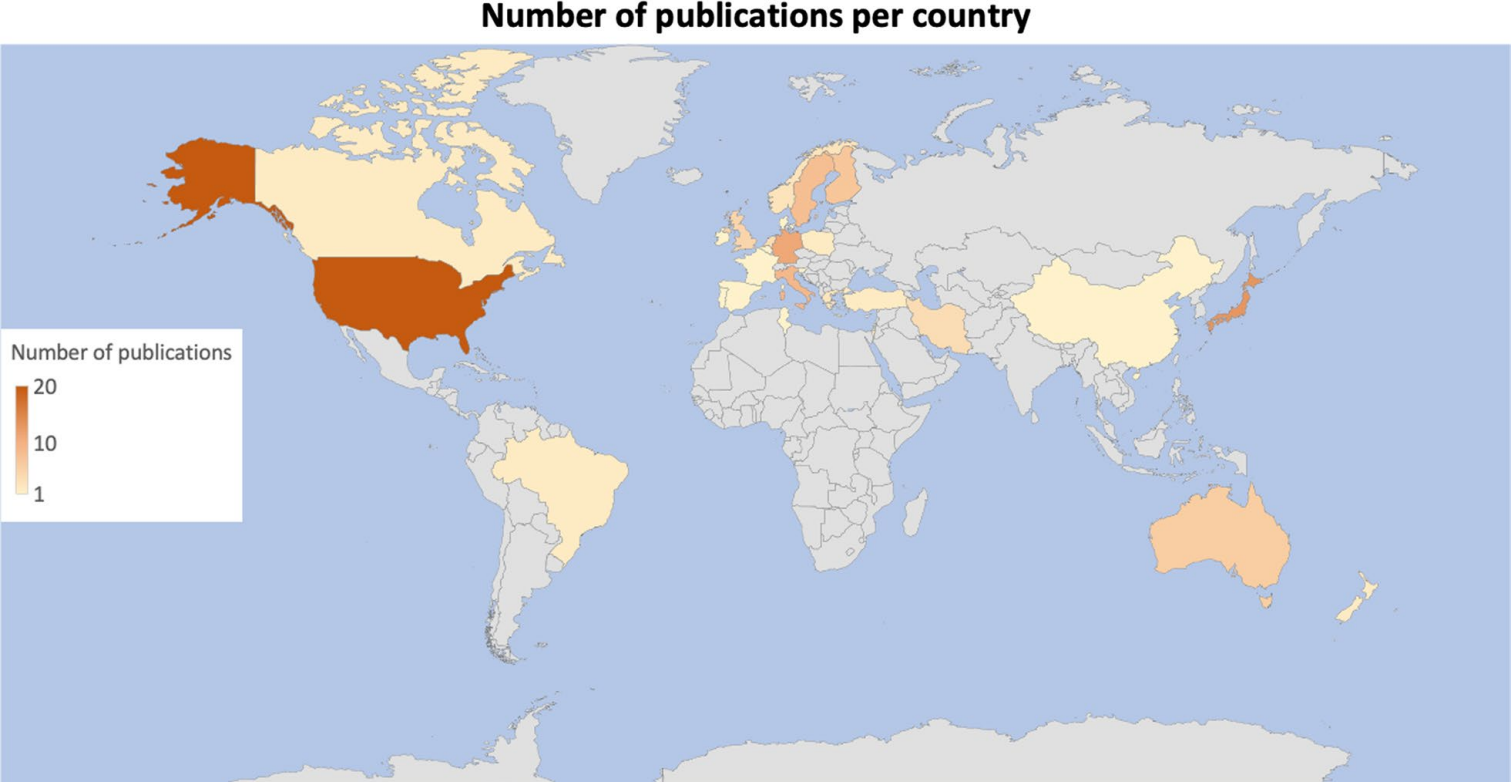
Distribution of research publications worldwide

Most of the studies (n = 20, 17.5%) were carried out in the United States, despite Europe -as a whole- publishing the majority of studies (n = 61, 53.5%). Other countries which gave a substantial contribution to the topic were Japan (n = 13, 11.5%), Germany (n = 11, 9.5%), Italy (n = 9, 8%) and Sweden (n = 8, 7%). Further details regarding all of the countries involved in this research context are reported in Fig. 2.

**Fig. 3:**
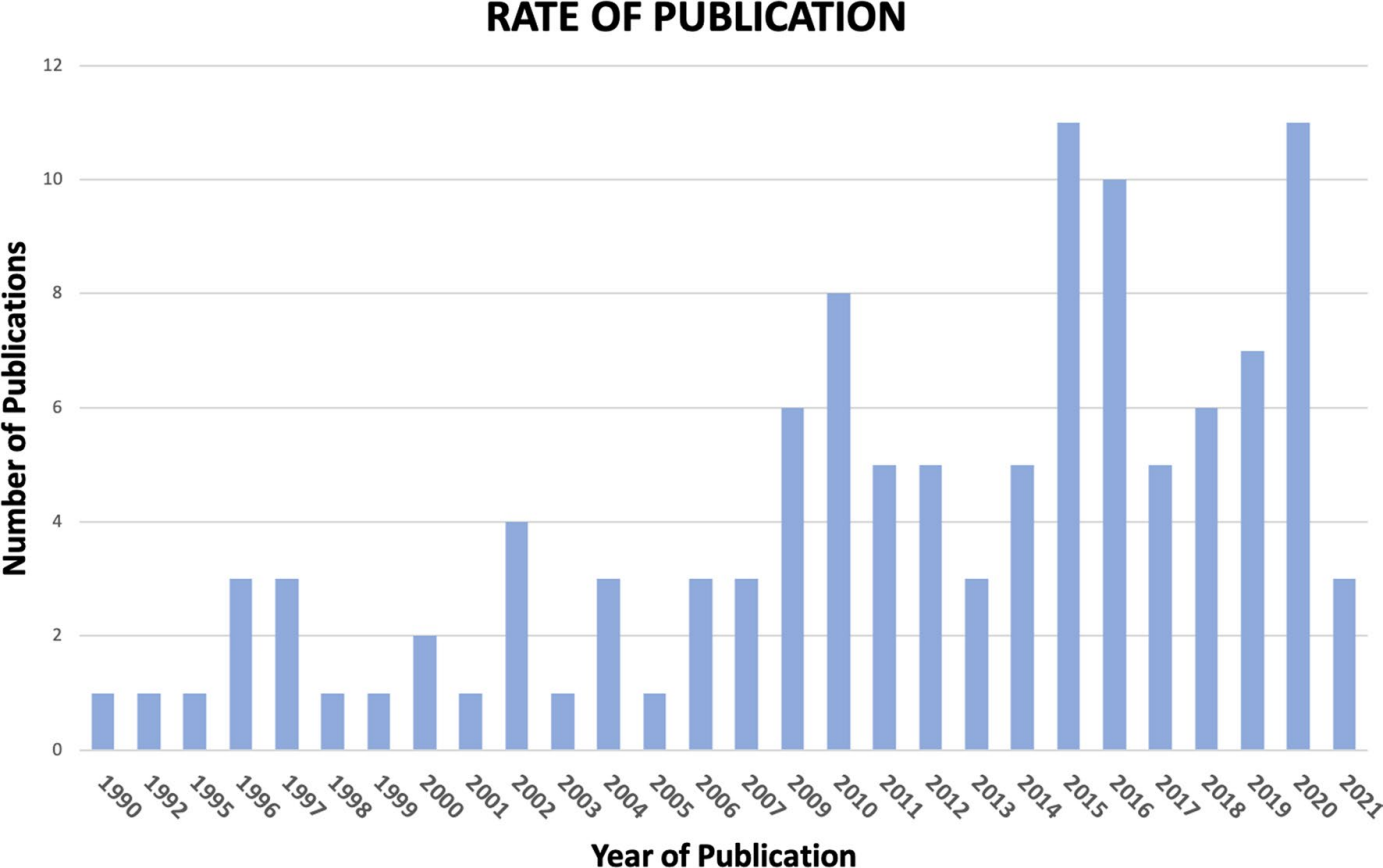
Distribution of publications over years since 1990

Number of publications increased over the years. However, most of them are concentrated in the time period 2015–2020. Specifically, in the years 2015 and 2020 eleven articles (n = 22, 19%) were published, followed by 2016 and 2019, respectively with 10 (9%) and 7 (6%) publications (Fig. 3). Approximately half of the included studies (52%) were published in sports medicine journals.

Furthermore, 70 studies (66%) considered in their sample only sports people, while the remaining 36 works compared them to people who didn’t practice sport.

#### Population

The included studies analyzed a total of 98,657 participants (mean: 941 ± 2889, median: 181, mode: 1, IQR: 566.5); 42,240 of the subjects were females (43%).

The mean age of the overall sample size was 22 ± 10.2 years (median: 20; mode: 20; min. age: 10; max. age: 56; IQR: 10).

68% of this population were professionals, 32% of athletes were classified as amateurs.

Specific details concerning sample characteristics are reported in Table 1.

**Table 1:** Characteristics of the overall sample of the included studies

Gender	N. of subjects
Female	42,240
Male	56,613

Age	Years
Mean (SD):	22.02 (10.2)
Median	20
Mode	20
Min–Max	10–56
IQR	10 (25–15)

Athletes	N. of studies
Amateurs	33
Professionals	71

Sample characteristics	N. of studies
Mixed sample	36
Only sports people:	70

Sample size	N. of subjects
Total	98,853
Mean (SD)	941.46 (2889.7)
Median	181
Mode	1
Min–Max	1–26,766
IQR	566.5 (633.5–67)

#### Sports

Several sports were considered, even if the vast majority of the studies investigated the combination of more than one activity as a risk factor for NS-LBP (42 works, representing 37% of the total). The most widely investigated sports were boating (7 studies, 6%), football, soccer, gymnastics, volleyball and running (5 studies, 4.5% each), basketball, tennis, dancing, swimming and skiing (4 studies, 3.5% each). Further examined sports and relative frequencies are specifically detailed in Additional file 3: Table C1 (Appendix C).

#### Context

Research was predominantly carried out by sports associations (53%), such as club teams, followed by clinical settings (clinic 10.5%, hospital 10.5% and private practice 7%) and colleges (19%).

Mostly, authors did not specify the athletes’ frequency of training (39 studies, 38%); in 17 works (16.5%) the sports people trained 5 times per week, in 16 cases (15.5%) 3 times per week, in 15 studies 4 times per week. Two studies investigated athletes who underwent training sessions every day of the week and twice per week (2%). Only one study (< 1%) considered one training session per week.

Almost half of the included studies (n = 51, 48.2%) were managed by physicians only, followed by physiotherapists (n = 10, 9.4%), chiropractors (n = 3, 3%) and sport science operators (n = 1, < 1%); in 23 studies (21.7%), the research was conducted by physicians and physiotherapists together. Further details are reported in Additional file 2: Fig. B2 (Appendix B).

In the assessment modality of NS-LBP, some differences are also present across studies. In 49% of cases (n = 52), a combination of interview, physical examination and pain-related questionnaires (scales) were administered by the personnel involved. In 38 studies (36%) only questionnaires were considered and in 11 articles (10%) only the physical evaluation was reported.

### Low back pain and sport relationship

All the reviewed studies aimed to investigate a possible association existing between sport and NS-LBP. The large majority of those works (n = 77, 67.5%) indicated sport as a possible predisposing factor. Conversely, 21 studies (18.5%) did not find any association and in 16 cases (14%) the association was referred to as “unclear”. On average, LBP rate was reported to be higher in athletes than in the general population, even though discrepancies have been found across studies. Different sport activities were found to have different impact on LBP development, with higher association in those involving repetitive or extreme loading of the spine (e.g., weightlifting, gymnastics), and in those leading to impulsive landing or impact forces (e.g., volleyball, basketball, football). Training frequency association with LBP was unclear, even though higher training volumes or periods of increased training were reported to be significantly related to LBP. Long-time athletes were also found to be more prone to LBP, while practicing more than one sport was not reported to be a significant predisposing factor.

## Discussion

### Summary of evidence

To the best of our knowledge, this is the first scoping review aimed at providing a comprehensive overview of the literature regarding the association between sport activities and NS-LBP. To date, two systematic reviews [6, 19] investigated prevalence and incidence of LBP in sports in general, highlighting important levels of methodological heterogeneity in the included studies. Thus, authors concluded that a complete synthesis of evidence was not possible up until then.

Furthermore, results show an increasing interest in this field, with a clear trend of growth in the number of publications over the last years. In this context, this ScR represents a proper systematic mapping of the current literature, potentially addressing clinical practice and future research [14].

### Low back pain and sport relationship

#### Differences in the investigated sports

One of the main themes that emerged from the results is represented by the investigated sports. As reported in the above section, the large majority of the studies considered the role of sport in general, including in their sample athletes who practice different activities. Similarly, some reviews [5, 6, 19, 20] focused on this topic as well, attempting to estimate an overall synthesis of the risk. Such a methodological choice provides information about the impact of the physical load. However, it prevents the understanding of the exact role of specific movement patterns related to the athletic gesture. This fact could represent an issue, since previous research confirmed a strong association between LBP and flexed, rotated and awkward positions of the lumbar spine [11, 21], or with repeated bending and twisting [22, 23]. Moreover, highly technical sports and those implying repetitive or extreme loading of the spine seemed to be at higher risk for LBP rather than endurance activities [19, 24]. For all these reasons, investigations on specific sports activities should be encouraged.

As outlined in the results section, research on this topic appears as a prerogative of western countries: Europe, United States and Canada provided by far the larger number of studies. Not surprisingly, the most investigated sport activities were football, swimming, volleyball, basketball and tennis, widely practiced in these countries [25]. Boating and dancing have also been widely considered, whereas other practices (e.g. martial arts, cricket) were investigated occasionally.

This fact has probable implications, since the focus of the research seems more influenced by the popularity of the activity, rather than by other variables such as the gestures, loading stress, solicitations and postures.

#### Differences in research methods

The included studies appeared heterogeneous with regards to the characteristics of training sessions, and an association with LBP was not clearly identified. Firstly, most of the studies did not provide any information concerning parameters such as duration, frequency and intensity of the practice. In addition, several studies presented many differences, although those parameters are considered crucial to define the dose–response rate for the risk assessment [26]. This aspect assumes even more relevance if we consider the supposed “U-shaped” relationship between physical activity and NS-LBP [27]. Such a relationship would address the detrimental effect of both low and strenuous levels of physical activity. However, higher training volumes seemed to increase LBP development risk in general [28].

Other relevant considerations are related to the study designs adopted by researchers. Cross-sectional investigations appear to be by far the most frequently used. From another point of view, data showed how 51% of the included studies (cross-sectional surveys and descriptive reports) were not primarily useful to assess the epidemiological relationship between sports and NS-LBP. Another 9% of the included works is represented by secondary research (reviews). Furthermore, only a minor part of studies compared athletes to non-athletes. As is known, only analytical cohort studies provide the best way to investigate such a relationship [29, 30]. For these reasons, the large percentage of studies (67.5%) reporting sports practice as a risk factor for NS-LBP should be considered carefully.

Higher homogeneity in LBP assessment methods is also needed, as they appeared different across studies, with only half of the works considering a multidimensional evaluation of the recruited subjects.

A relatively large number of the included records were case reports. These were predominantly descriptions of particular painful conditions, originally classified as NS-LBP and successively revealed to be related to other areas (e.g. hip problems, ileo-tibial syndrome) or to a specific type of LBP (mostly stress fractures or spondylolysis). As is well known, LBP assessment often represents a diagnostic challenge [31] and these reports provide valuable cues to help clinicians in the difficult evaluation of such painful conditions [32, 33].

### Implications for clinical practice and research

According to the results of the current review, the relationship between sports and NS-LBP is still far from being demonstrated. Recent systematic reviews considered different sports as a whole, underlining the role of duration, intensity and frequency of sessions as possible risk factors [5, 6, 19, 20]; from another point of view, several studies highlighted biomechanics and specific athletic gestures as important for LBP onset [34–36]. It is our assumption that clinicians should consider both of these hypotheses during their assessment, without forgetting the distinctive clinical features of each single subject.

Secondly, a multidisciplinary approach seems to be essential in NS-LBP [37]. At the moment, most of the research is mainly led by physicians, sometimes in combination with physiotherapists. Other practitioners such as sport science operators, manual therapists and chiropractors contributed occasionally, although their role could be of considerable importance [38–41]. For the same reasons, sports associations and clubs should be equipped with a team of clinicians, preferably experts in specific LBP clinical management. As previously discussed, the remarkable presence of case reports in literature confirms the above-mentioned difficulties during the assessment process [31].

As a direct consequence of the results obtained in this study, research should be more directed towards analytical studies. In particular, high-quality prospective double-parallel cohort designs are preferably needed to provide the best possible evaluation of the risk in the association “sports - NS-LBP” [30]. Case–control studies could represent an option, as long as their major exposure to biases is considered [42].

Furthermore, research focus should be oriented in two distinctive directions: on the one hand, more evidence is needed on single activities, especially regarding movement patterns and their connection to LBP onset. On the other hand, the characteristics of training should be further investigated. In this regard, it is of crucial importance that future research be more similar in terms of exposure to physical efforts and that relative parameters (e.g. duration, intensity and frequency of training) also be well documented [11].

Lastly, some sports such as martial arts, baseball, hockey and weightlifting are studied little and require more in-depth research.

### Limitations

As this study consists in a comprehensive mapping of the literature, our search strategy might have lost some pertinent records. Thus, results and relative conclusions could have been influenced.

Another possible limitation can be related to the term “athlete”. In practice, we accepted the definition reported by the authors of each work. However, some differences are probably present among the included studies.

For the sake of synthesis, we decided to exclude narrative reviews, letters and editorials from the selection of the studies. Although these formats do not produce real evidence, they represent a form of contribution to literature that is not present in our reporting.

## Conclusions

This review mapped the literature regarding the potential relationships between sports and NS-LBP. Results showed an increasing interest in this topic over the last years.

Currently, research is more centered on sport as a whole, although some activities are more investigated than others. At the moment, research methods are extremely varied, thus more high-quality, standardized observational research may focus on specific disciplines and relative training modalities to properly contribute to research and clinical practice.

## Supplementary Information

**Additional file 1**: Search Strategy used in Pubmed (Medline).

**Additional file 2**: **Fig. B1**. Study design distribution of the included studies. **Fig. B2**. Graphic distribution of the personnel involved in the research.

**Additional file 3**: **Table C1**. Distribution of investigated sport activities across the included studies.

## Data Availability

The datasets used and/or analyzed during the current study are available from the corresponding author on reasonable request.

## Abbreviations

NS-LBP: Non-specific low back pain
ScR: Scoping review
LBP: Low back pain
JBI: Joanna Briggs Institute
PRISMA-ScR: Preferred reporting items for systematic reviews and meta-analysis extension for scoping reviews
SD: Standard deviation
IQR: Interquartile ranges
