# Supplementary material for: Sport and non-specific low back pain in athletes: a scoping review
Source: BMC Sports Sci Med Rehabil. 2022 Dec 23;14:216. doi: 10.1186/s13102-022-00609-9 (PMC9789643; doi:10.1186/s13102-022-00609-9)
Supplement: Supplementary file 1 — Additional file 1: Search Strategy used in Pubmed (Medline). [file 13102_2022_609_MOESM1_ESM.docx]

**APPENDIX A.** Search Strategy used in Pubmed (Medline)

**(((((((((((sport*[Title/Abstract]) OR (athlet*[Title/Abstract])) OR (football[Title/Abstract])) OR (volleyball[Title/Abstract])) OR (tennis[Title/Abstract])) OR (basket*[Title/Abstract])) OR (running*[Title/Abstract])) OR (soccer[Title/Abstract])) OR (cycling*[Title/Abstract])) OR (gymnastic*[Title/Abstract])) OR (sports[MeSH Terms])) AND ((((((((((("low back pain"[Title]) OR ("spinal pain"[Title])) OR ("back pain"[Title])) OR ("backache"[Title])) OR ("lumbago"[Title])) OR ("non-specific low back pain"[Title])) OR ("non specific low back pain"[Title])) OR (low back pain[Title])) OR ("lower back pain"[Title])) OR ("low back ache"[Title])) OR ("chronic low back pain"[Title]))**
