# Supplementary material for: Sport and non-specific low back pain in athletes: a scoping review
Source: BMC Sports Sci Med Rehabil. 2022 Dec 23;14:216. doi: 10.1186/s13102-022-00609-9 (PMC9789643; doi:10.1186/s13102-022-00609-9)
Supplement: Supplementary file 2 — Additional file 2: Fig. B1. Study design distribution of the included studies. Fig. B2. Graphic distribution of the personnel involved in the research. [file 13102_2022_609_MOESM2_ESM.docx]

**Appendix B**

| *Figure B1. Study design distribution of the included studies* |
| --- |
| *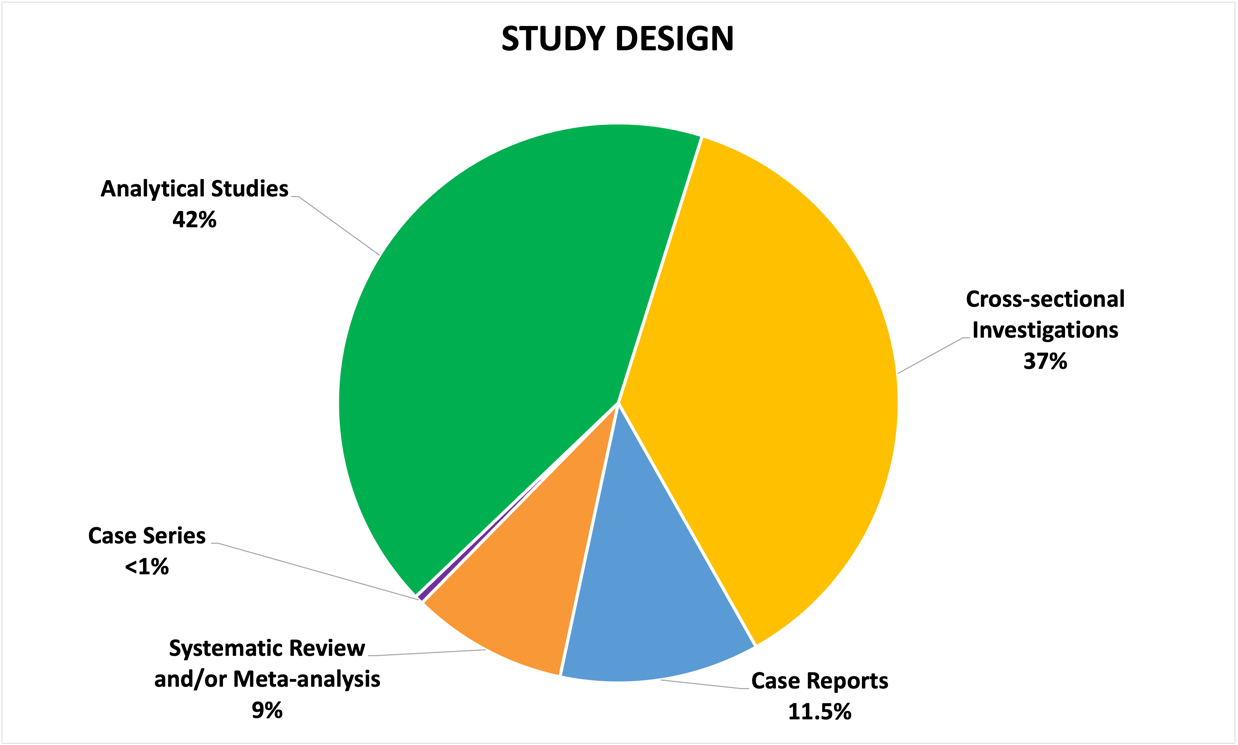* |

| *Figure B2. Graphic distribution of the personnel involved in the research* |
| --- |
| 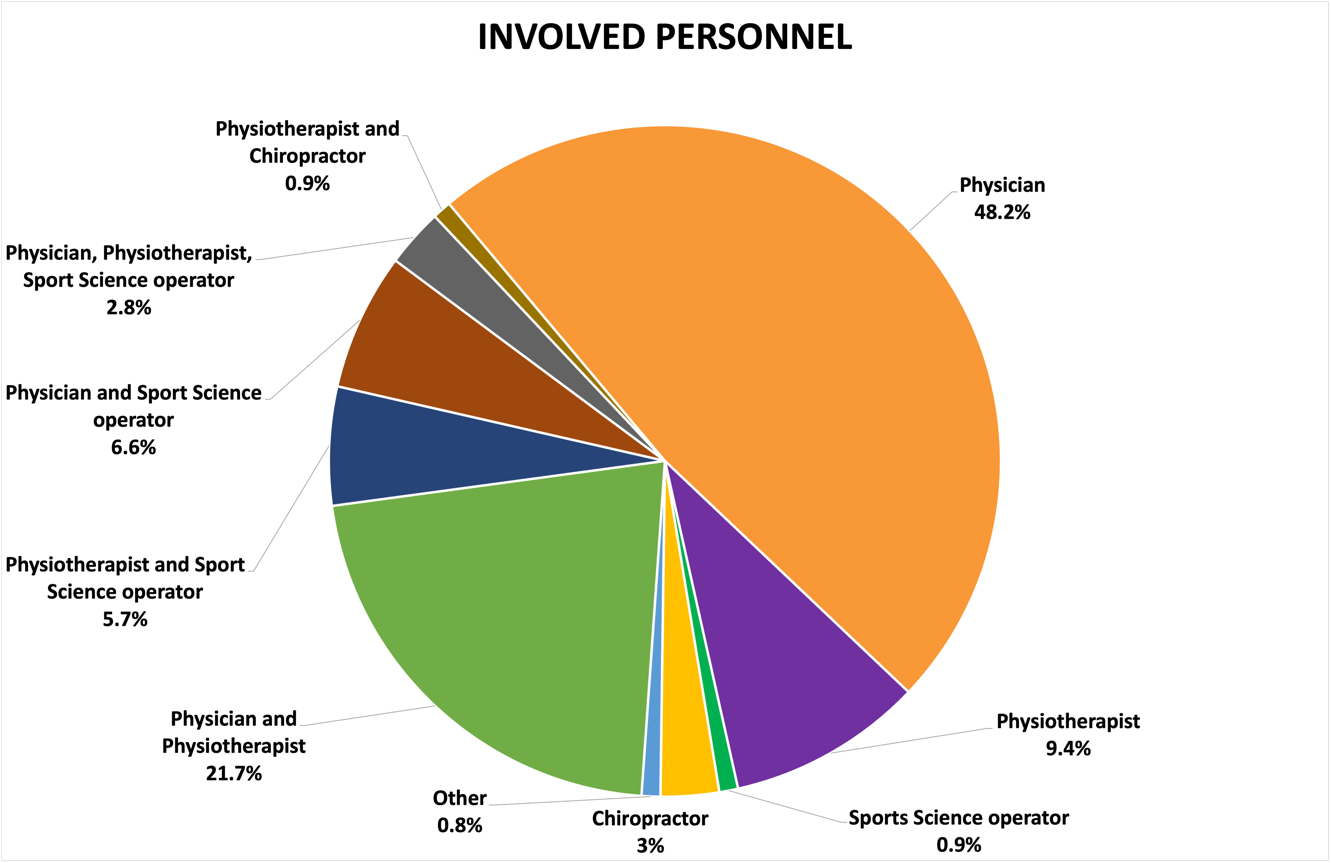 |
