# Supplementary material for: Sport and non-specific low back pain in athletes: a scoping review
Source: BMC Sports Sci Med Rehabil. 2022 Dec 23;14:216. doi: 10.1186/s13102-022-00609-9 (PMC9789643; doi:10.1186/s13102-022-00609-9)
Supplement: Supplementary file 3 — Additional file 3: Table C1. Distribution of investigated sport activities across the included studies. [file 13102_2022_609_MOESM3_ESM.docx]

**Appendix C**

| *Table C1. Distribution of investigated sport activities across the included studies* | | |
| --- | --- | --- |
| **Sport** | **N. of studies** | **Percentage %** |
| Sport in general | 42 | 36.8 |
| Boating | 7 | 6.1 |
| Soccer, Volleyball, Running, Football, Gymnastics | 5 | 4.4 each |
| Tennis, Basketball, Dancing, Swimming, Skiing | 4 | 3.5 each |
| Cycling | 3 | 2.6 |
| Athletics, Weightlifting, Golf, Cricket | 2 | 1.8 each |
| Karate, Jiujitsu, Beach volley, Judo, Triathlon, Baseball, Padel, Hockey | 1 | 0.9 each |
